# Microscopic Investigations in Cases of Sterility

**Published:** 1870-02

**Authors:** B. H. Cheney


ARTICLE V. — *Microscopic Investigation in Cases of Sterility.* By B. H. CHENEY, M.D.

"*Ut alia sic alia facere possumus.*" — PROVERBIUM POPULI AMERICANI.

IN the January (1869) number of the *New York Medical Journal*, is an article by Dr. J. Marion Sims on "The Microscope as an Aid in the Diagnosis and Treatment of Sterility." This article was previously read by its learned author at the meeting of the medical society of the county of New York, December 7, 1868.

I wish to say, at the outset, that I entertain the greatest respect for Dr. Sims, as one whose deservedly high reputation reflects credit upon the profession, and upon our country. I also realize that it may perhaps seem presumptuous in so humble a member of the profession as myself to criticise him. Still, there are things in the paper above referred to, concerning which not only every medical man ought to be capable of forming a decided individual opinion, but also which should be noticed by the profession at large.

The object, in substance, of Dr. Sims' paper, is to recommend, in investigating cases of sterility, the microscopic examination of the semen of the husband; this semen being obtained with a speculum from the vagina of the wife, as soon as may be after coition. This examination is thus instituted for the purpose of settling "three questions which must be decided before any case of sterility can be treated understandingly," viz.:

"1. We must be sure that we have semen with spermatozoa."

"2. We must ascertain if the spermatozoa enter the utero-cervical canal."

"3. We must determine whether the secretions of this canal are favorable or not to the vitality of the spermatozoa."

Critics and reviewers are often accused of misrepresentation, or of leading to false impressions by quoting only a sentence or two here and there from the work or article under consideration, omitting the context, which might perhaps put a different construction upon the whole matter. To clear myself from any such imputation, and as some of your readers may not have seen the paper of Dr. Sims, I will quote from it that portion to which especial reference is here made. Let Dr. Sims, then, speak for himself:

"To find out all at once, and with the least delay and trouble, I usually say to the husband or wife, as it may be, 'It is very important before instituting any treatment, to be sure that the seminal fluid enters the neck of the womb, for without this, conception is impossible. We must also ascertain if the uterine secretions kill the semen; if so, a certain treatment will be necessary. If you will, then, send your wife here, or come with her any day, five or six hours after coition, it will be easy to settle these points at once.' In nineteen cases out of twenty, the wife presents herself the next day. The speculum is introduced, (and when I say the speculum, I always mean the one that bears my name), and some vaginal mucus is removed by the syringe, and placed on an object-glass. Then some cervical mucus is drawn out and placed on another object-glass. These two specimens are then examined under the microscope. If we find spermatozoa, well and good; but if we find none, neither in the vaginal, nor cervical mucus, our fears are at once aroused. What, then, is to be done? I simply say that I am not quite satisfied with the examination, and would like to see the wife again, at some future time, under the same circumstances. But, suppose we find no spermatozoa on this second examination? Then two questions immediately arise: either, that there are no spermatozoa, or that the semen has all passed off before the case came under observation. Sometimes the semen is all instantly thrown off by the vagina, and then it would not do to pronounce the husband sterile till we are sure of a specimen of his semen for investigation. If I fail to satisfy myself on this point, I then explain the possibility of the semen all passing off, in the act of rising and dressing, and show the absolute necessity of making the examination half an hour or so," (so=less?) "after coition, and before the erect posture is assumed. When the subject is presented in this plain, practical manner, and treated seriously, no man or woman of sense could oppose it; and, with me, it has never, in a single instance, been objected to."*

Now, I am not, by any means, disposed to be prudish, or hypercritical in medical or scientific matters. Of course, "to the pure all things are pure;" and if the maxim that "the end sanctifies the means," be ever justifiable, it is so in circumstances and considerations affecting the health, the well-being, or the happiness of any human being.

But still, it would seem that to adopt such a procedure as that recommended above, is to go a little too far. The doctor, whoever he may be, earnest, enthusiastic, waits in an adjoining apartment, armed with speculum, sponge, syringe and microscope, while the husband and wife retire to celebrate the rites of hymen under the kindly favoring auspices of stern science. The act consummated, the disciple of Æsculapius, who is, for the nonce, the representative of science, and who, let us hope, though conscious of his proud mission, bears himself with becoming dignity and meekness, is ushered by the husband into the chamber where the wife is lying, still flushed and warm from the marital embrace, "not yet having assumed the erect posture." He introduces the speculum with the comforting words, "Madam, I will soon tell you why you don't have a baby." Perfect silence reigns. The expectant couple, yearning for parental joys, await in trembling hope the oracle, which is to bid them try again, or give it up as hopeless. The man of science looks, examines, tests, and finally gives — his opinion. For, after all, it is simply an opinion.

I would respectfully submit the question: ought not any competent physician to be able to determine, at least so far as to serve as a basis and guide for a rational therapy, whether a given case of sterility be curable, or beyond treatment, by a separate examination of the husband and wife, without resorting to the procedure recommended by Dr. Sims? The three questions, the settlement of which he very correctly insists upon as essential for the proper understanding and treatment of the case, viz.: as to the presence of spermatozoa in the semen; as to the possibility or impossibility of the spermatozoa entering the utero-cervical canal; and

* *New York Journal of Medicine*, January, 1869, pp. 400–401.

as to the character of the vaginal and utero-cervical secretions being favorable or not to the vitality of the spermatozoa; — these three questions, which are all he claims to determine by his method, can certainly be about as well decided by a separate examination of the husband and wife.

That Dr. Sims should advocate such a measure, is certainly cause for surprise; second only to it, is the wonder we feel when we read that his proposition for such an examination has, with him, "never, in a single instance, been objected to." For one would naturally think that most husbands and wives would scarcely consider the possible, and, at the best, uncertain result, a sufficient compensation for the means.

I would not be "old-fogyish" in conservatism, and am certainly far more disposed to welcome and adopt, than to criticise, or, least of all, ridicule, any thing which may conduce to the advancement of science, or the welfare of humanity. But it surely seems that if this procedure had not the indorsement and prestige of a name so deservedly high as that of Dr. Sims, it would be too ridiculous and indecent to be noticed.

